# Chimeras of Bet v 1 and Api g 1 reveal heterogeneous IgE responses in patients with birch pollen allergy

**DOI:** 10.1016/j.jaci.2013.12.1073

**Published:** 2014-07

**Authors:** Barbara Gepp, Nina Lengger, Merima Bublin, Wolfgang Hemmer, Heimo Breiteneder, Christian Radauer

**Affiliations:** a Department of Pathophysiology and Allergy Research, Center for Pathophysiology, Infectiology and Immunology, Medical University of Vienna, Vienna, Austria; b Floridsdorfer Allergiezentrum, Vienna, Austria

**Keywords:** Bet v 1.0101, Api g 1.0101, patient-specific IgE repertoire, chimera-based technology, IgE epitope mapping, birch pollen allergy

## Abstract

**Background:**

Characterization of IgE-binding epitopes of allergens and determination of their patient-specific relevance is crucial for the diagnosis and treatment of allergy.

**Objective:**

We sought to assess the contribution of specific surface areas of the major birch pollen allergen Bet v 1.0101 to binding IgE of individual patients.

**Methods:**

Four distinct areas of Bet v 1 representing in total 81% of its surface were grafted onto the scaffold of its homolog, Api g 1.0101, to yield the chimeras Api-Bet-1 to Api-Bet-4. The chimeras were expressed in *Escherichia coli* and purified. IgE binding of 64 sera from Bet v 1–sensitized subjects with birch pollen allergy was determined by using direct ELISA. Specificity was assessed by means of inhibition ELISA.

**Results:**

rApi g 1.0101, Api-Bet-1, Api-Bet-2, Api-Bet-3, and Api-Bet-4 bound IgE from 44%, 89%, 80%, 78%, and 48% of the patients, respectively. By comparing the amount of IgE binding to the chimeras and to rApi g 1.0101, 81%, 70%, 75%, and 45% of the patients showed significantly enhanced IgE binding to Api-Bet-1, Api-Bet-2, Api-Bet-3, and Api-Bet-4, respectively. The minority (8%) of the sera revealed enhanced IgE binding exclusively to a single chimera, whereas 31% showed increased IgE binding to all 4 chimeras compared with rApi g 1.0101. The chimeras inhibited up to 70% of IgE binding to rBet v 1.0101, confirming the specific IgE recognition of the grafted regions.

**Conclusion:**

The Bet v 1–specific IgE response is polyclonal, and epitopes are spread across the entire Bet v 1 surface. Furthermore, the IgE recognition profile of Bet v 1 is highly patient specific.

Birch is one of the main elicitors of pollinosis in Europe.^1^ More than 98% of patients with birch pollen allergy from Austria, Finland, and Sweden are sensitized to the major birch pollen allergen Bet v 1,^2^ which belongs to the pathogenesis-related 10 family of plant pathogenesis-related proteins.^3^ Additionally, more than 70% of patients with birch pollen allergy have adverse reactions to certain plant foods.^4^ This cross-reactivity is caused by sensitization to Bet v 1 and binding of Bet v 1–specific IgE to homologous plant food allergens. Bet v 1–related proteins have been identified as major allergens in apple (Mal d 1), celery (Api g 1), cherry (Pru av 1), and carrot (Dau c 1), among others.^5-8^

Until now, little has been known about the nature of IgE-binding epitopes of Bet v 1 and related plant food allergens. Epitope mapping is crucial to understand immune responses to allergens and allergen cross-reactivity among homologous proteins. Furthermore, knowledge about pivotal IgE-binding regions provides the basic information required for the design of safe and effective reagents used for allergen-specific immunotherapy, the only curative and specific approach in the treatment of allergy.^9^

It was shown that IgE binding to Bet v 1 was highly dependent on the protein's native conformation.^10,11^ Thus the analysis of IgE-binding epitopes of Bet v 1 represents a challenging task. Thus far, only 1 epitope was indirectly determined by means of the cocrystallization of Bet v 1.0112 and the Fab fragment of a murine mAb capable of blocking IgE binding to Bet v 1 by 40%.^11^ This epitope covered the P-loop, a highly conserved region among pathogenesis-related 10 family members.^12^ Furthermore, the contribution of the P-loop to IgE binding of Bet v 1 was proved by means of site-directed mutagenesis of Bet v 1 and Pru av 1. Exchange of Glu45 in both proteins reduced IgE binding for most patients' sera.^13,14^ The existence of high and low IgE binding isoallergens of Bet v 1 and the generation of hypoallergenic mutants led to the definition of further key residues important for IgE binding to Bet v 1 and Mal d 1.^15-17^ Another strategy to identify epitopes is based on mimicking the epitope in its interaction with IgE by short peptides selected from random-peptide libraries. This so-called mimotope technology was applied to identify preferred IgE-binding regions of Bet v 1.^18,19^ Engineering of chimeric proteins of Bet v 1 and homologous proteins represents a further approach for investigating B-cell epitopes. By using epitope grafting, 3 IgE-binding regions important for cross-reactivity between Bet v 1 and Mal d 1 were examined.^20-22^

Because the diversity of the IgE response to Bet v 1 among individual patients with birch pollen allergy has never been investigated in detail, we aimed to determine the patient-specific IgE recognition profile of a large group of patients. We generated 4 chimeras of Bet v 1.0101 and its low-allergenic, nonsensitizing homolog Api g 1.0101 from celeriac.^23-25^ On the basis of the known crystal structures of Bet v 1^26^ and Api g 1,^27^ 4 selected Bet v 1–specific portions covering the major part of the molecular surface were grafted onto the Api g 1 scaffold.

## Methods

### Patients and control subjects

In a retrospective study 64 residual serum samples of Austrian Bet v 1–sensitized patients with birch pollen allergy drawn during routine diagnosis at the Floridsdorfer Allergiezentrum, Vienna, Austria, were included (see Table E1 in this article's Online Repository at www.jacionline.org). The patients underwent no interventions related to the study. The use of anonymized serum samples and clinical records without obtaining written consent of the patients was approved by the ethics committee of the Medical University of Vienna (approval no. 718/2010).

Patients were selected on the basis of a typical case history of birch pollen allergy, positive skin prick test responses to birch pollen, and/or *in vitro* IgE detection to rBet v 1 or birch pollen extract (≥0.35 kU_A_/L; ImmunoCAP, Thermo-Fisher, Uppsala, Sweden). The average age of the patients was 34 years (range, 7-79 years). The sex distribution was balanced, with 56% female and 44% male patients. History of food allergy to common birch pollen–associated plant foods was assessed based on questioning by an experienced allergist. Fifty-two percent (n = 33) had allergic symptoms after ingestion of plant foods, with a single patient reporting celery allergy. Twenty-two percent (n = 14) did not report food allergies, and for the rest (n = 17), these data were not available. As a negative control, sera from 7 nonallergic patients without histories of type I allergy to common allergen sources were included.

### Design of the Api g 1–Bet v 1 chimeras

**Fig 1:**
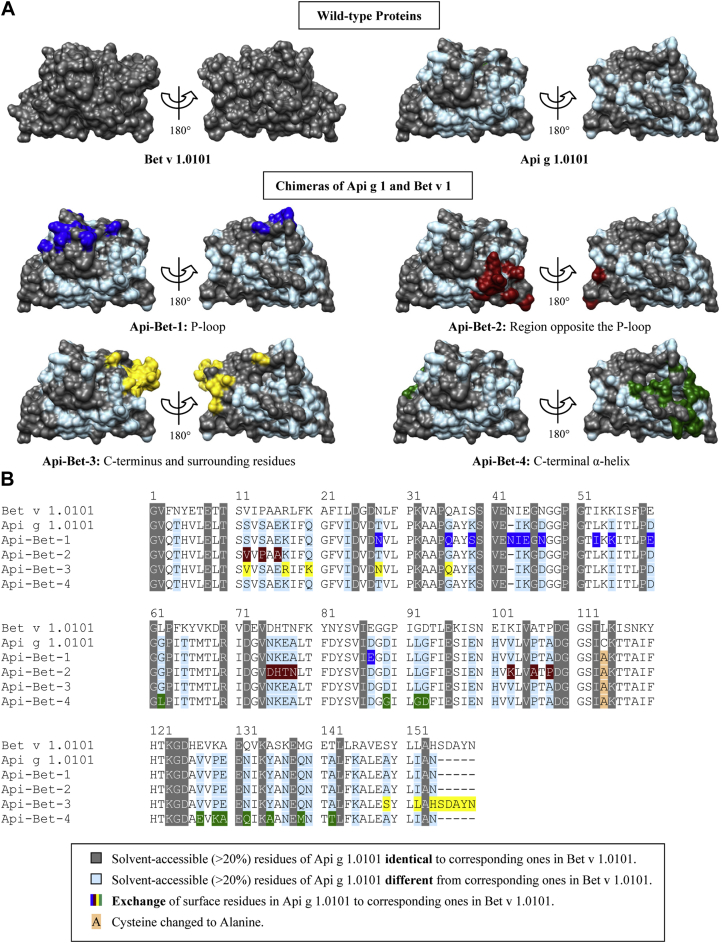
Sequence and structural comparison of the chimeras. **A,** Front and back views (rotated by 180° around a vertical axis) of the parent molecules and the chimeras are depicted. *Colors* indicating mutated residues were mapped onto the Api g 1 surface. The models were prepared with UCSF Chimera.^28^**B,** Multiple sequence alignment of proteins.

Chimeric proteins of Bet v 1.0101 and its homolog Api g 1.0101 were generated to investigate IgE binding to defined Bet v 1.0101–specific surface areas. Grafting of Bet v 1–specific surface areas onto the Api g 1.0101 scaffold was achieved by replacing Api g 1.0101–specific solvent-accessible (>20%) residues by corresponding Bet v 1.0101–specific residues (Fig 1). We generated the chimeric protein Api-Bet-1 by grafting Glu45, the central residue of the previously identified P-loop epitope, and surrounding residues, identified by using UCSF Chimera,^28^ onto Api g 1.0101. The region opposite the P-loop (Api-Bet-2), the C-terminus and surrounding residues (Api-Bet-3), and the C-terminal α-helix (Api-Bet-4) of Bet v 1.0101 were grafted in the same manner to generate spatially well-distributed Bet v 1.0101–specific surface areas on Api g 1.0101.

### Cloning, expression and purification, and physicochemical analysis of the recombinant proteins

Production and analysis of the recombinant proteins was performed as described in the Methods section in this article's Online Repository at www.jacionline.org.

### IgE ELISA

For direct ELISA, microtiter plates (Maxisorp; Nalge Nunc International, Roskilde, Denmark) were coated overnight at 4°C with 1 μg/mL individual chimeric proteins, a mixture of all chimeras (1 μg/mL each), and rApi g 1.0101 or rBet v 1.0101, respectively, in 50 mmol/L sodium carbonate buffer, pH 9.6. After blocking of nonspecific binding sites, sera (1:10 dilution) were incubated in duplicates overnight at 4°C. Specific IgE was detected by using an alkaline phosphatase–conjugated mouse anti-human IgE mAb (BD Pharmingen, San Jose, Calif), followed by color development with Sigma FAST p-nitrophenyl phosphate tablets (Sigma-Aldrich, St Louis, Mo) and measurement of the absorbance at 405 nm.

OD values were measured at several time points. For each serum, the measurement with an OD of approximately 1.0 for Bet v 1.0101 was normalized to a 1-hour substrate incubation period after subtracting the OD values of the buffer controls (see Table E2 in this article's Online Repository at www.jacionline.org). Comparison of measurements at different times proved that the OD values increased with time in a linear fashion (data not shown). Hence normalized OD values were roughly proportional to allergen-specific IgE concentrations.

Individual sera from 7 nonallergic donors were included as negative controls. Normalized OD values exceeding the mean negative control value by more than 3 SDs were considered positive.

For each serum, specific IgE binding to the grafted regions of each chimera was assessed by calculating the difference of the OD values of the chimera and rApi g 1.0101. The difference was considered positive if it exceeded 3 times the SD of the negative control value.

### ELISA inhibition

For inhibition ELISA, coating, blocking, and detection were performed, as described above. Either Bet v 1.0101 or the chimeras were coated to the solid phase. In inhibition assays, in which rBet v 1.0101 was coated, IgG was removed in advance by means of incubation of prediluted sera on an anti-human IgG (BD Pharmingen)–coated plate. Sera were diluted 30- to 100-fold. Inhibition was performed by preincubating diluted sera with 10-fold serial dilutions from 0.01 to 100 μg/mL of the individual chimeric proteins, a mixture of all chimeras, and rApi g 1.0101 or rBet v 1.0101, respectively, before they were applied to the plates.

For cross-inhibition between the chimeras, all chimeras, rApi g 1.0101, and rBet v 1.0101 (1 μg/mL) were coated to the solid phase and incubated with patients' sera (diluted 10- to 60-fold). The supernatants were transferred to a second plate, which was coated with all 4 chimeras or buffer only. IgE binding to the second plate was detected, as described above.

Inhibition values were calculated as follows:

$$\text{Inhibition}\;\left[\text{\%}\right]=\left(1-{\text{OD}}_{\text{inhibited}}/{\text{OD}}_{\text{noninhibited}}\right)\times 100.$$

### ELISA with Bet v 1–specific mAbs

Binding of Bet v 1–specific mAbs to rBet v 1, rApi g 1, and the chimeras was tested by using ELISA, as described in the Methods section in this article's Online Repository.

### Statistical analyses

The Friedman test (α = .05) was performed to test whether the amount of IgE binding to each of the 4 chimeras differed significantly from that to rApi g 1.0101. The relationship between the number of chimeras recognized better than rApi g 1.0101 and the amount of rBet v 1–specific IgE present in patients' sera was analyzed by performing Spearman correlation (α = .05).

## Results

### Biochemical characterization of the recombinant proteins

The structural integrity of the recombinant proteins was confirmed by means of circular dichroism spectroscopy, mass spectrometry, and ELISA with Bet v 1–specific mAbs, as described in the Results section in this article's Online Repository at www.jacionline.org.

### IgE-binding profiles of Bet v 1 are highly patient specific

IgE-binding capacities of rBet v 1.0101, rApi g 1.0101, and the chimeras were determined by means of ELISA. All 64 tested sera displayed rBet v 1–specific IgE, whereas only 44% of the sera bound to rApi g 1 (Table I). Total IgE-binding capacities of all 4 chimeras were significantly higher than that of rApi g 1 (*P* < .001; median OD for rApi g 1, 0.028; median ODs for the chimeras, 0.076-0.222; see Fig E3 in this article's Online Repository at www.jacionline.org).

For each serum, specific IgE binding to the grafted regions of each chimera was assessed by calculating the difference of the ELISA OD values of the chimera and the template rApi g 1.0101. The grafted regions of Api-Bet-1, Api-Bet-2, and Api-Bet-3 were recognized by 70% to 81% of the sera, whereas only 45% recognized Api-Bet-4 (Table I).

**Fig 2:**
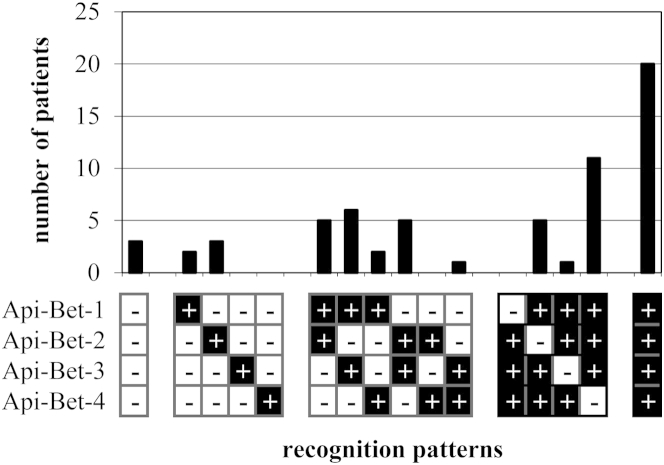
Patient-specific patterns of IgE binding to the grafted regions of chimeric proteins. IgE binding of Bet v 1–sensitized patients' sera (n = 64) was determined by means of IgE ELISA. The OD values obtained with rApi g 1 were subtracted, and significantly positive values were counted.

Patients were categorized according to their binding patterns to evaluate individual IgE recognition profiles (Fig 2). Interestingly, only 5 (8%) of 64 sera exclusively recognized the grafted regions of a single chimeric protein. Furthermore, 19 and 17 (30% and 27%, respectively) sera showed increased IgE binding to 2 or 3 chimeras, respectively, compared with rApi g 1.0101. The highest number of patients possessed IgE directed to all 4 grafted regions (20/64 [31%]). Only 3 (5%) sera did not bind to any grafted region.

### Inhibition ELISA confirms IgE specificity for the grafted areas

**Fig 3:**
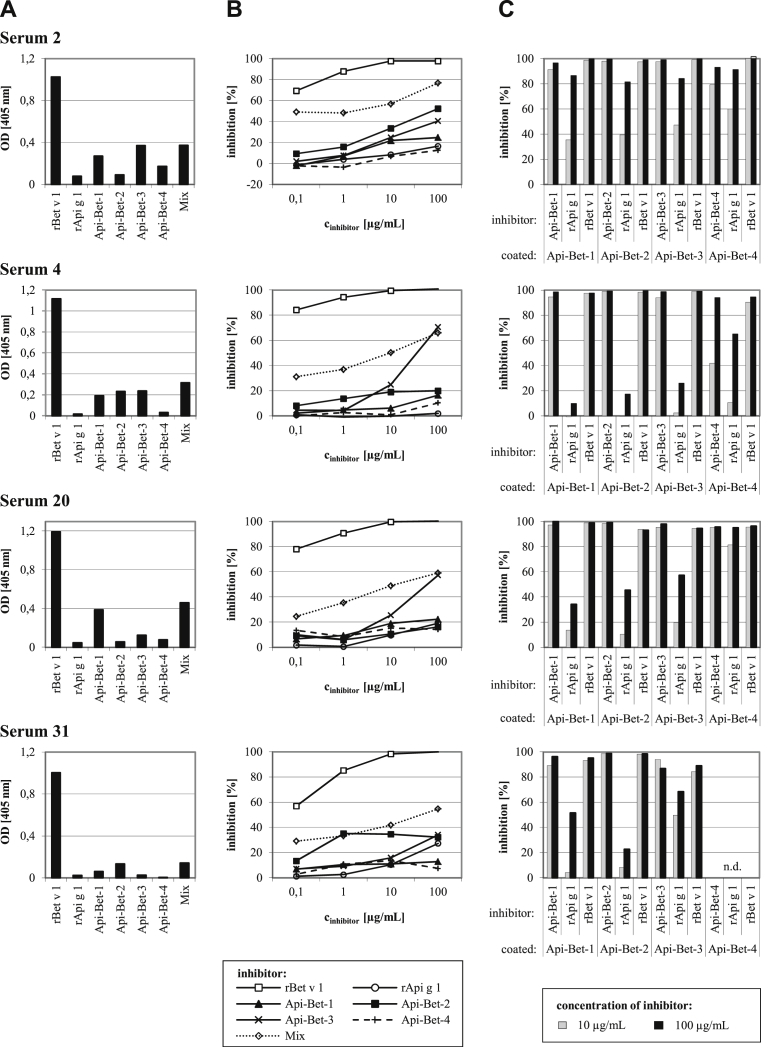
ELISA data of 4 representative patients. **A,** Direct ELISA showing IgE binding to rBet v 1.0101, rApi g 1.0101, the chimeras, and a mix of all chimeras (nonnormalized OD values). **B** and **C,** Inhibition of IgE binding to immobilized rBet v 1.0101 (Fig 3, *B*) or the chimeras (Fig 3, *C*) by means of preincubation with rBet v 1.0101 (positive control), rApi g 1.0101, and the chimeras. *n.d*., Not done.

Data of 4 representative sera are depicted in Fig 3. Direct ELISA data (Fig 3, *A*) show the highly patient-specific IgE-binding patterns to the chimeric proteins. The percentage of Bet v 1–specific IgE that bound to the chimeras was tested by using an inhibition ELISA (Fig 3, *B*) in which rBet v 1.0101 was coated to the solid phase. Self-inhibition of rBet v 1.0101 was complete at inhibitor concentrations of 10 μg/mL. In most cases the chimeras did not reach saturating inhibition, even at 100 μg/mL. Maximum inhibition values ranged from 7% to 71%, depending on the serum and the inhibitor protein. A mixture of all 4 chimeras inhibited IgE binding to Bet v 1 by 55% to 77%. In contrast, rApi g 1 inhibited IgE binding to rBet v 1 by only 2% to 27%.

The 4 chimeras were coated to the solid phase to examine the percentage of chimera-specific IgE that bound to the grafted areas (Fig 3, *C*). Inhibition was performed with rBet v 1.0101, rApi g 1.0101, and the immobilized chimeras. In all cases almost complete inhibition of IgE binding to the chimeras by rBet v 1.0101 was observed. The extent to which rApi g 1.0101 inhibited IgE binding to the chimeras showed large differences between the tested sera. Inhibitions with 3 sera (4, 20, and 31) yielded percentages between 10% and 68%, with the exception of a single high value. In contrast, inhibitions with serum 2 resulted in high extents of inhibition (82% to 91%) for all 4 chimeras, indicating low percentages of IgE binding to the grafted areas.

Furthermore, ELISA inhibitions were performed in which IgE binding to each chimera was inhibited by all other chimeras (see Fig E5, *B*, in this article's Online Repository at www.jacionline.org). We observed partial cross-reactivity between Api-Bet-1 and Api-Bet-4, as well as between Api-Bet-2 and Api-Bet-3, for some sera.

### The amount of Bet v 1–specific IgE correlates with the number of chimeras recognized

The number of chimeras to which IgE binding to the grafted region was detected and the amount of Bet v 1–specific IgE in patients' sera showed a significant correlation (*r* = 0.35, *P* = .01; see Fig E4 in this article's Online Repository at www.jacionline.org). Comparing the OD values of the Bet v 1–specific IgE ELISA after 1 hour yielded a median OD of 1.03 for sera that recognized no chimeras, whereas this value was 1.98 and 2.39 for sera binding to 3 or all 4 chimeras, respectively.

## Discussion

Thus far, little is known about the distribution of IgE-binding epitopes on the surface of the major birch pollen allergen Bet v 1. We grafted defined Bet v 1 surface areas onto the structurally homologous celery allergen Api g 1.0101, which has a much lower capacity to bind IgE from patients with birch pollen allergy. We then used these chimeric proteins to analyze IgE binding to the grafted areas for a large group of patients with birch pollen allergy. A similar approach was used to investigate IgE binding to the P-loop^20^ and other relevant single amino acid residues of Bet v 1^21,22^ or other allergens.^29-31^ However, we are the first to analyze IgE binding to a large portion of the solvent-exposed surface area of Bet v 1.0101.

The combined mutated residues of all 4 chimeric allergens, including residues conserved between Bet v 1.0101 and Api g 1.0101, comprised more than 80% of the molecular surface of Bet v 1.0101. To obtain reliable data, we ensured that the recombinant proteins folded correctly. We checked the secondary structures of the chimeras using circular dichroism spectroscopy and obtained spectra highly similar to that of rApi g 1 (see Fig E1, *A*, in this article's Online Repository at www.jacionline.org). Furthermore, we performed an ELISA with 2 Bet v 1–specific mAbs that bound to rBet v 1 and Api-Bet-1 but not to the other chimeras and rApi g 1 (see Fig E2 in this article's Online Repository at www.jacionline.org). Moreover, a Bet v 1–specific, recombinant, human single-chain variable antibody fragment (manuscript in preparation) exclusively bound to rBet v 1 and Api-Bet-3. These experiments proved that Api-Bet-1 and Api-Bet-3 contained single Bet v 1–like regions on their surfaces, which were responsible for their specific antibody-binding abilities. The correct fold of the chimeras was further substantiated by the fact that the amounts of IgE binding to all chimeras were equal or greater than the amounts of rApi g 1–specific IgE in 62 of 64 sera (see Table E3 in this article's Online Repository at www.jacionline.org).

Several studies aiming to map IgE-binding epitopes on Bet v 1.0101 were performed. The first IgE-binding epitope on Bet v 1 was located by means of crystallization of an antibody-antigen complex and comprised an area covering the P-loop.^11^ The crucial role of this conserved region for IgE binding was confirmed in several studies.^11,20,32^ Furthermore, the fact that an mAb binding to this epitope was able to inhibit specific IgE binding by approximately 40% supported the concept that a few epitopes dominated the IgE response to Bet v 1.^14^

The P-loop of Api g 1.0101 is different because it harbors a positively charged lysine instead of a negatively charged glutamic acid at the corresponding position in Bet v 1.0101. We investigated in detail IgE binding to the P-loop of Bet v 1.0101 by replacing 11 amino acids of Api g 1.0101 by the corresponding Bet v 1.0101–derived residues (Fig 1). The Api g 1 derivative generated was termed Api-Bet-1, and 81% of the patients with birch pollen allergy showed higher IgE binding to this chimera than to rApi g 1.0101 (Table I). Surprisingly, only 2 of 64 patients exclusively recognized Api-Bet-1, indicating that the area around the P-loop is by far not the only region important for IgE binding to Bet v 1.0101 (Fig 2). This prediction is corroborated by the fact that, contrary to Api g 1.0101, the isoallergen Api g 1.0201, which comprises a P-loop similar to Bet v 1.0101,^23^ has a low IgE-binding capacity.

In contrast to Bet v 1.0101, Api g 1.0101 has a C-terminus shortened by 5 residues. In Api-Bet-3 these residues were added and another 8 amino acids were mutated to obtain a Bet v 1–specific area around the C-terminus (Fig 1). Contrary to Neudecker et al,^13^ who showed that removing the C-terminal residues 155-159 from Pru av 1.0101 did not affect IgE reactivity, we observed an increased recognition of Api-Bet-3 compared with rApi g 1.0101 for 75% of the sera (Table I). Furthermore, Api-Bet-3 exhibited the highest IgE binding capacity (see Fig E3) and was able to inhibit IgE binding to rBet v 1 by up to 70% (Fig 3, *B*).

For Api-Bet-4, 11 amino acids were mutated in Api g 1.0101 to create a Bet v 1–specific area around the C-terminal α helix (Fig 1). Compared with the other chimeras, Api-Bet-4 bound the lowest amount of IgE, and only 45% of the patients showed enhanced IgE binding to this chimera compared with rApi g 1.0101. In a recent study^22^ an rBet v 1–specific IgE antibody was selected from a phage library constructed from IgE-encoding cDNAs isolated from Bet v 1–sensitized patients. This antibody bound to the C-terminal helix of Bet v 1, but not to Mal d 1, which was also proved by grafting the C-terminal helix of Bet v 1 onto Mal d 1. However, the significance of this epitope was not tested with patients' sera.

In our study we showed that all the chimeras, each of which was bearing a distinct Bet v 1–specific surface area, bound IgE from a high percentage of patients' sera. In line with our data, previous studies showed that the entire surface of a protein is potentially antigenic.^33^ Interestingly, 31% of patients with birch pollen allergy recognized all 4 grafted areas, indicating that the immune response to Bet v 1.0101 is highly polyclonal. Nevertheless, we observed a total of 12 different recognition profiles in our patient sample, with the number of bound chimeras between 0 and 4 (Fig 2).

Consistent with our results, a high patient-to-patient variation was also observed when investigating IgE binding to the P-loop mutants of Pru av 1 and Api g 1.0101.^13,23,34^ Furthermore, Holm et al^20^ observed patient-specific IgE repertoires by grafting of a Bet v 1–specific epitope containing the P-loop onto Mal d 1. Moreover, IgE binding to various Bet v 1 mutants displayed divergent recognition patterns,^16,35^ and high heterogeneity of IgE specificity could also be observed by comparing IgE binding to Bet v 1 and homologous food proteins.^19^ However, most of these studies were performed with small patient groups.

In a recent study of allergen-specific IgE from patients sensitized to the major house dust mite allergen Der p 2, it was shown that the complexity of the allergic patients' IgE repertoire correlated with the serum concentration of allergen-specific IgE.^36^ In accordance with this finding, we revealed that the amount of Bet v 1–specific IgE present in patients' sera correlated with the number of chimeras recognized, a value representing the complexity of the IgE epitope repertoire (see Fig E4).

Reduction of IgE cross-linking on the surfaces of mast cells or basophils by vaccine components during specific immunotherapy is crucial for preventing severe side effects. Therefore characterization of IgE-binding epitopes is of paramount importance for developing artificial hypoallergens or peptide vaccines for safer and more effective immunotherapy. Mapping of IgE epitopes by using a chimera-based approach offers the possibility of analyzing a defined area (eg, a single IgE epitope) of an allergen with polyclonal patients' sera. Thus this technology might be used as diagnostic tool to determine the patient-specific response to defined epitopes of a major allergen or to cross-reactive homologs. In addition, this will pave the way for a patient-tailored epitope-based therapy.

Taken together, this study demonstrates that it is possible to graft defined areas of a major allergen onto a low IgE-binding homolog to evaluate IgE binding to the grafted region. Furthermore, investigation of a large group of patients with birch pollen allergy showed that the repertoire of Bet v 1–specific IgE is highly patient specific and polyclonal. A single major epitope on Bet v 1.0101 important for all patients with birch pollen allergy does not exist, and relevant IgE-binding epitopes are located across the entire surface of Bet v 1.0101.

**Key messages**

• The Bet v 1–specific IgE response is polyclonal, and the recognition profile is highly patient specific.
• The existence of a single major IgE epitope on Bet v 1 can be excluded.
• The IgE epitopes are distributed across the entire surface of Bet v 1.

## Figures and Tables

**Table I:** Frequencies of IgE binding among patients with birch pollen allergy (n = 64) to rBet v 1, rApi g 1, and the chimeras determined by means of ELISA

	Frequencies of recognition	Frequencies of IgE binding to grafted regions∗
rBet v 1	100%	100%
rApi g 1	44%	—
Api-Bet-1	89%	81%
Api-Bet-2	80%	70%
Api-Bet-3	78%	75%
Api-Bet-4	48%	45%

∗ Percentage of patients with IgE binding significantly increased compared with rApi g 1.

## Supplementary data

Online Repository Data

Fig E1

Fig E2

Fig E3

Fig E4

Fig E5
