# Supplementary material for: Chimeras of Bet v 1 and Api g 1 reveal heterogeneous IgE responses in patients with birch pollen allergy
Source: J Allergy Clin Immunol. 2014 Jul;134(1):188–94. doi: 10.1016/j.jaci.2013.12.1073 (PMC4085476; doi:10.1016/j.jaci.2013.12.1073)
Supplement: Online Repository Data [file mmc1.docx]

**ONLINE REPOSITORY**

**Chimeras of Bet v 1 and Api g 1 reveal heterogeneous IgE responses in birch pollen-allergic patients**

Barbara Gepp MSc^1^, Nina Lengger^1^, Merima Bublin PhD^1^, Wolfgang Hemmer PhD^2^, Heimo Breiteneder PhD^1^, Christian Radauer PhD^1^

^1^Department of Pathophysiology and Allergy Research, Center for Pathophysiology, Infectiology and Immunology, Medical University of Vienna, Vienna, Austria

^2^Floridsdorfer Allergiezentrum, Vienna, Austria

Corresponding author:

Heimo Breiteneder PhD

Department of Pathophysiology and Allergy Research, Medical University of Vienna,

Währinger Gürtel 18-20, 1090 Vienna, Austria.

Tel.: +43 1 40400 5102; Fax: +43 1 40400 5130.

E-mail address: heimo.breiteneder@meduniwien.ac.at

This study was supported by grants SFB-F4608 (HB) and P22559-B11 (CR) from the Austrian Science Fund.**METHODS**

**Cloning, Expression and Purification of Recombinant Proteins**

# Expression constructs were generated by inserting synthetic codon-optimized DNA molecules (Eurofines MWG Operon, Ebersberg, Germany) into the *Escherichia coli* vectors pET28a(+) (Novagen, Madison, WI, USA) for Bet v 1.0101, Api g 1.0101, Api-Bet-1 and Api-Bet-4 (NcoI/EcoRI used) and pET32a(+) (Novagen) for Api-Bet-2 and Api-Bet-3 (BglII/EcoRI used). Proteins expressed in the latter vector yield fusion proteins with an N-terminal 6x histidine tag and thioredoxin. For removal of the His-tagged thioredoxin an oligonucleotide encoding a PreScission Protease (GE Healthcare, Little Chalfont, UK) cleavage site (5’-LeuGluValLeuPheGln/GlyPro-3’) was inserted directly upstream of the Api-Bet-2 and -3 genes between the NcoI and BglII restriction sites.

# All chimeras, Bet v 1.0101 and Api g 1.0101 were expressed in freshly transformed *Escherichia coli* BL21 [DE3] (Novagen, Madison, WI) and grown at 30° C in LB-medium supplemented with 25 mg/L kanamycin (Bet v 1.0101, Api g 1.0101, Api-Bet-1 and Api-Bet-4) or at 20°C in LB-medium supplemented with 100 mg/L ampicillin (Api-Bet-2 and -3). Protein expression was induced by addition of 1 mM isopropyl β-D-1-thiogalactopyranoside at an OD_600_ of 0.8. After overnight expression, the cells were harvested by centrifugation. The pellets were resuspended in 50 mM Tris/HCl pH 8.0, 10 mM EDTA, 10 mM DTT supplemented with protease inhibitor tablets (Roche Diagnostics, Mannheim, Germany) for rBet v 1.0101, rApi g 1.0101, Api-Bet-1 and -4 or 50 mM Tris/HCl pH 8.0, 300 mM NaCl supplemented with protease inhibitor tablets for Api-Bet-2 and -3 and lysis was performed using a French Pressure Cell (SLM Aminco, Rochester, NY, USA).

# Purification of rBet v 1.0101, rApi g 1.0101, Api-Bet-1 and -4 was achieved by hydrophobic interaction chromatography (Phenyl Sepharose; GE Healthcare, Little Chalfont, UK), anion exchange chromatography (Q-Sepharose; GE Healthcare) and size exclusion chromatography (Sephacryl S-200; GE Healthcare). For Api-Bet-2 and -3, the major part of the fusion proteins remained in the pellet. The soluble fraction was directly loaded onto a Ni^2+^-based immobilized metal ion affinity chromatography column (IMAC-Sepharose; GE Healthcare). The insoluble fraction was denatured with 50 mM Tris/HCl pH 8.0, 8 M Urea, 300 mM NaCl, loaded onto the IMAC-column and renatured in 50 mM Tris/HCl, 300 mM NaCl, 5% glycerol by decreasing the urea concentration from 8 M to 0 M within 120 minutes at 0.8 mL/min. Elution was achieved with 100 mM EDTA pH 8,0. Purification of Api-Bet-2 and -3 was obtained after cleavage of the fusion proteins with PreScission Protease (GE Healthcare), removal of thioredoxin with IMAC-Sepharose (GE Healthcare) and removal of PreScission Protease with affinity (GSTrap, GE Healthcare) or size exclusion chromatography (Sephacryl S-200; GE Healthcare). The purified proteins were dialyzed against 10 mM sodium phosphate buffer pH 7.4 and stored at -20°C.

**Physicochemical analysis of the recombinant proteins**

Purity of the recombinant proteins was analyzed by SDS-PAGE and Coomassie Brilliant Blue R-250 staining. The identity was checked by Matrix-assisted laser desorption/ionization-time of flight (MALDI-TOF) mass spectrometry (Bruker Daltonics, Bremen, Germany). Secondary structures were checked by circular dichroism spectroscopy and recorded in 10 mM sodium phosphate buffer pH 7.4 at 0.2 mg/mL in a 0.1-cm quartz cuvette using a J-810 spectopolarimeter (Jasco, Easton, MD, USA).

**ELISA with monoclonal anti-Bet v 1 antibodies**

Microtiter plates (Maxisorp, Nalge Nunc International, Roskilde, Denmark) were coated overnight at 4°C using 2 µg/mL recombinant protein in 50 mM sodium carbonate buffer, pH 9.6. After blocking of non-specific binding sites, cell culture supernatants of BIP 1 and BIP 4^1^ (1:10 dilution) were incubated in duplicates overnight at 4°C. Bound antibodies were detected by a 1:5000 diluted alkaline phosphatase-conjugated rabbit anti-mouse IgM+IgG antibody (Jackson ImmunoResearch, Suffolk, UK) followed by color development using Sigma FAST p-nitrophenyl phosphate tablets (Sigma-Aldrich, St. Loise, MO, USA) and measurement of the absorbance at 405nm.

**Results**

**Biochemical characterization of the recombinant proteins**

To confirm structural integrity of the recombinant proteins, the presence of secondary structure elements was analyzed by CD-spectroscopy. rBet v 1.0101, rApi g 1.0101 and the chimeric proteins showed nearly identical CD-spectra typical for folded proteins with mixed α-β structures (Fig. E1A). Api-Bet-2 and Api-Bet-3 were obtained after denaturing purification and in vitro refolding. Nevertheless, their CD-spectra were identical to those of protein preparations obtained after native purification (Fig. E1B).

Furthermore, MALDI-TOF mass spectrometry was used to show the integrity of the recombinant proteins. The molecular masses of the chimeric proteins were measured as follows (theoretical mass is given in parentheses): rBet v 1.0101 (17439) 17439, rApi g 1.0101 (16189) 16190, Api-Bet-1 (16358) 16359, Api-Bet-2 (16461) 16465, Api-Bet-3 (17157) 17154, Api-Bet-4 (16115) 16113. These values confirm the correct cleavage of Api-Bet-2 and Api-Bet-3 from thioredoxin and prove that the initial methionine of Api-Bet-1 and Api-Bet-4 was removed.

In order to confirm the structural integrity of the chimeric proteins, we performed an ELISA with the Bet v 1-specific monoclonal antibodies BIP 1 and BIP 4^1^ (Fig. E2). Both antibodies bound to rBet v 1 and Api-Bet-1, but not to rApi g 1 and the other chimeras.

**REFERENCES**

1. Jarolim E, Rumpold H, Endler AT, Ebner H, Breitenbach M, Scheiner O, et al. IgE and IgG antibodies of patients with allergy to birch pollen as tools to define the allergen profile of Betula verrucosa. Allergy 1989; 44:385-95.

**TABLES**

**TABLE E1:** Clinical data of the patients included in this study.

| **#** | **Age** | **Sex** | **Food allergies** | **Positive skin prick test^a^** | **Total IgE [kU/l]** | **Bet v 1 CAP [kU/l]** |
| --- | --- | --- | --- | --- | --- | --- |
| 1 | 17 | m | n.k. | bi ash gr hdm | 96 | 35.4 |
| 2 | 34 | m | apple, pear, nuts | bi hdm | 310 | 77.5 |
| 3 | 51 | f | n.k. | bi gr hdm ca do | 29 | 11.2 |
| 4 | 24 | m | apple, nuts, kiwi | bi ash gr | 605 | 82.5 |
| 5 | 7 | m | kiwi | bi ash gr | 290 | >100 |
| 6 | 25 | m | n.k. | bi ash gr rag pla | 85 | 14.0 |
| 7 | 13 | f | n.k. | bi gr mug ash hdm ca do alt | 1100 | 35.4 |
| 8 | 25 | m | no | bi gr hdm ca do | 57 | 4.2 |
| 9 | 42 | m | n.k. | bi hdm | 66 | 45.1 |
| 10 | 62 | f | no | bi | 82 | 4.2 |
| 11 | 46 | m | apple | bi ash gr | 102 | 11.2 |
| 12 | 34 | m | no | bi ash gr pla hdm | 135 | 14.0 |
| 13 | 29 | f | no | bi gr | 82 | 8.4 |
| 14 | 16 | m | apple, citrus | bi gr ca alt | 245 | 14.0 |
| 15 | 40 | f | no | bi gr mug rag hdm | 315 | >100 |
| 16 | 16 | m | n.k. | bi ash gr | 300 | 92.5 |
| 17 | 61 | f | n.k. | bi ca | 860 | >100 |
| 18 | 43 | f | n.k. | bi mug pla | 21 | 7.0 |
| 19 | 34 | m | no | bi ash gr rag pla ca do alt | 250 | 45.1 |
| 20 | 55 | f | apple, pear, nuts, soja | bi | 154 | 87.5 |
| 21 | 50 | f | no | bi hdm | 130 | 16.8 |
| 22 | 24 | m | n.k. | bi ash | 31 | 22.4 |
| 23 | 36 | m | n.k. | bi ash pla | 120 | 19.1 |
| 24 | 38 | f | n.k. | bi gr mug ca ho | 925 | 87.5 |
| 25 | 33 | f | n.k. | bi alt | 14 | 7.0 |
| 26 | 10 | m | n.k. | bi ash mug gr hdm ca | 157 | 12.6 |
| 27 | 8 | m | apple, cherry | bi gr hdm alt | 66 | 35.4 |
| 28 | 34 | f | apple, pear, stone fruit, nuts | bi gr hdm ca | 246 | 41.9 |
| 29 | 47 | f | apple, peach, kiwi | bi gr ca do hdm | 272 | >100 |
| 30 | 18 | f | no | bi gr mug hdm ca | 148 | 9.8 |
| 31 | 79 | m | apple | bi ash gr mug ca do pla | 810 | 45.1 |
| 32 | 30 | f | no | bi gr | 98 | 25.6 |
| 33 | 30 | f | spinach | bi gr ash rap pla mug | 240 | 35.4 |
| 34 | 29 | f | apple, carrot | bi gr pla mug hdm | 63 | 25.6 |
| 35 | 41 | f | n.k. | bi | 50 | 22.4 |
| 36 | 15 | f | apricot, peanut | bi gr ash pla hdm | 803 | 25.6 |
| 37 | 38 | m | n.k. | bi gr ca do | 1330 | 32.1 |
| 38 | 13 | f | n.k. | bi ash gr mug rag | 910 | >100 |
| 39 | 36 | m | apple | bi ash gr hdm | 92 | 52.5 |
| 40 | 34 | f | n.k. | bi ash gr hdm | 165 | 62.5 |
| 41 | 27 | f | apple, pear, stone fruit, nuts | bi ash gr mug rag | 380 | >100 |
| 42 | 22 | f | no | bi | 56 | 25.6 |
| 43 | 37 | f | nuts | bi | 420 | >100 |
| 44 | 24 | f | apple | bi gr pla hdm | 345 | 15.4 |
| 45 | 21 | m | apple, nuts | bi gr hdm ca ho | 435 | 87.5 |
| 46 | 35 | m | apple, nuts, carrot | n.k. | 1700 | >100**^b^** |
| 47 | 30 | f | nuts | n.k. | 386 | 17.51-50**^b^** |
| 48 | 30 | f | apple, nuts | bi gr alt | 38 | 3.51-17.5 |
| 49 | 28 | m | apple, peach | n.k. | 36 | 5.05**^b^** |
| 50 | 32 | f | apple, peach | n.k. | 70 | 16 |
| 51 | 31 | f | apple, nuts, peach | bi | 4155 | 47.7 |
| 52 | 30 | f | apple, nuts, peach | n.k. | 31 | 8.57**^b^** |
| 53 | 38 | m | no | n.k. | 444 | 76.4 |
| 54 | 28 | m | no | bi alt | 47 | 8.15**^b^** |
| 55 | 28 | f | apple, peach, cherry, carrot, nuts, jackfruit, tempeh | n.k. | 256 | 11.0 |
| 56 | 24 | f | no | n.k. | 92 | 1.37**^b^** |
| 57 | 41 | m | apple, peach, cherry, carrot, jackfruit | n.k. | 57 | 2.5 |
| 58 | 40 | m | no | n.k. | 83 | 9.74**^b^** |
| 59 | 34 | m | nuts, apple | n.k. | 217 | 50**^b^** |
| 60 | 49 | f | apple, pear, apricot, peach, cherry, nuts | n.k. | 50 | 14.0 |
| 61 | 40 | f | apple, pear | n.k. | 70 | 3.7 |
| 62 | 63 | f | apple, celery, carrot, citrus, egg white | n.k. | 158 | 2.3 |
| 63 | 67 | f | apple, nuts, peach, apricot | n.k. | 34 | 12.3 |
| 64 | 43 | m | apple, nuts, melon, banana | n.k. | 559 | 25.1 |

**^a^ bi:** birch, **gr**: grass, **mug**: mugwort, **pla**: plantain, **rag**: ragweed, **rap**: rape, **ca**: cat, **do**: dog, **ho**: horse, **hdm**: house dust mite, **alt**: Alternaria

**^b^** CAP to birch pollen

**n.k.**: not known

**TABLE E2:** Raw and normalized IgE-ELISA OD-values of sera from all allergic patients’ (1-64) and non-allergic individuals’ (NHS1-NHS7) sera included in this study.

| **Measured ELISA OD values (raw data) after subtracting buffer control values** | | | | | | |  | **Normalized ELISA OD/h** | | | | | |
| --- | --- | --- | --- | --- | --- | --- | --- | --- | --- | --- | --- | --- | --- |
| **Serum #** | **rBet v 1** | **rApi g 1** | **Api-Bet-1** | **Api-Bet-2** | **Api-Bet-3** | **Api-Bet-4** | **Substrate incubation period [min]** | **rBet v 1** | **rApi g 1** | **Api-Bet-1** | **Api-Bet-2** | **Api-Bet-3** | **Api-Bet-4** |
| 1 | 0,856 | 0,039 | 0,204 | 0,060 | 0,183 | 0,113 | 10 | 5.134 | 0.237 | 1.222 | 0.362 | 1.097 | 0.677 |
| 2 | 1,250 | 0,083 | 0,308 | 0,210 | 0,423 | 0,198 | 15 | 5.001 | 0.334 | 1.231 | 0.840 | 1.693 | 0.793 |
| 3 | 0,936 | -0,001 | 0,014 | 0,118 | 0,308 | 0,018 | 30 | 1.872 | -0.001 | 0.028 | 0.237 | 0.616 | 0.036 |
| 4 | 1,617 | 0,006 | 0,303 | 0,277 | 0,322 | 0,050 | 15 | 6.469 | 0.024 | 1.211 | 1.110 | 1.288 | 0.198 |
| 5 | 0,897 | 0,008 | 0,048 | 0,253 | 0,481 | 0,050 | 7 | 7.690 | 0.072 | 0.409 | 2.165 | 4.123 | 0.432 |
| 6 | 0,820 | 0,086 | 0,280 | 0,076 | 0,113 | 0,165 | 30 | 1.641 | 0.172 | 0.560 | 0.153 | 0.226 | 0.330 |
| 7 | 0,913 | 0,007 | 0,189 | 0,113 | 0,107 | 0,014 | 15 | 3.651 | 0.029 | 0.757 | 0.451 | 0.429 | 0.055 |
| 8 | 0,675 | 0,024 | 0,038 | 0,044 | 0,056 | 0,076 | 60 | 0.675 | 0.024 | 0.038 | 0.044 | 0.056 | 0.076 |
| 9 | 0,878 | 0,118 | 0,328 | 0,148 | 0,293 | 0,319 | 25 | 2.106 | 0.283 | 0.787 | 0.354 | 0.703 | 0.767 |
| 10 | 0,893 | 0,003 | 0,337 | 0,073 | 0,141 | 0,013 | 60 | 0.893 | 0.003 | 0.337 | 0.073 | 0.141 | 0.013 |
| 11 | 1,016 | 0,014 | 0,093 | 0,098 | 0,021 | 0,025 | 30 | 2.032 | 0.028 | 0.187 | 0.195 | 0.041 | 0.051 |
| 12 | 1,812 | 0,054 | 0,332 | 0,037 | 0,113 | 0,112 | 7 | 15.532 | 0.461 | 2.844 | 0.316 | 0.970 | 0.964 |
| 13 | 0,856 | 0,018 | 0,015 | 0,090 | 0,029 | 0,033 | 40 | 1.284 | 0.028 | 0.023 | 0.135 | 0.043 | 0.049 |
| 14 | 0,929 | 0,067 | 0,068 | 0,076 | 0,161 | 0,200 | 30 | 1.857 | 0.134 | 0.137 | 0.152 | 0.322 | 0.400 |
| 15 | 0,915 | 0,006 | 0,010 | 0,072 | 0,125 | 0,012 | 10 | 5.489 | 0.037 | 0.060 | 0.434 | 0.752 | 0.069 |
| 16 | 1,145 | 0,017 | 0,389 | 0,067 | 0,119 | 0,132 | 10 | 6.868 | 0.105 | 2.334 | 0.403 | 0.714 | 0.792 |
| 17 | 1,045 | 0,038 | 0,224 | 0,048 | 0,121 | 0,084 | 7 | 8.953 | 0.330 | 1.918 | 0.409 | 1.039 | 0.718 |
| 18 | 0,817 | 0,039 | 0,127 | 0,127 | 0,186 | 0,100 | 45 | 1.090 | 0.052 | 0.169 | 0.170 | 0.249 | 0.134 |
| 19 | 0,974 | 0,003 | 0,173 | 0,046 | 0,015 | 0,003 | 20 | 2.922 | 0.009 | 0.519 | 0.138 | 0.046 | 0.010 |
| 20 | 1,067 | 0,018 | 0,327 | 0,113 | 0,124 | 0,106 | 15 | 4.270 | 0.070 | 1.309 | 0.451 | 0.495 | 0.423 |
| 21 | 0,915 | 0,035 | 0,158 | 0,137 | 0,248 | 0,108 | 25 | 2.195 | 0.083 | 0.380 | 0.328 | 0.596 | 0.260 |
| 22 | 0,901 | -0,003 | -0,004 | 0,127 | 0,085 | -0,001 | 20 | 2.702 | -0.009 | -0.013 | 0.381 | 0.255 | -0.002 |
| 23 | 0,939 | 0,002 | 0,056 | 0,059 | 0,011 | -0,002 | 25 | 2.254 | 0.005 | 0.133 | 0.142 | 0.026 | -0.005 |
| 24 | 0,754 | 0,028 | 0,125 | 0,103 | 0,244 | 0,134 | 20 | 2.263 | 0.083 | 0.376 | 0.308 | 0.731 | 0.401 |
| 25 | 0,771 | 0,021 | 0,018 | 0,004 | 0,034 | 0,006 | 45 | 1.027 | 0.028 | 0.024 | 0.006 | 0.046 | 0.008 |
| 26 | 0,897 | 0,014 | 0,024 | 0,092 | 0,180 | 0,030 | 35 | 1.538 | 0.024 | 0.041 | 0.158 | 0.308 | 0.052 |
| 27 | 0,981 | 0,009 | 0,162 | 0,115 | 0,207 | 0,068 | 25 | 2.355 | 0.022 | 0.388 | 0.275 | 0.498 | 0.162 |
| 28 | 1,045 | 0,019 | 0,113 | 0,166 | 0,276 | 0,045 | 20 | 3.135 | 0.057 | 0.339 | 0.498 | 0.828 | 0.136 |
| 29 | 1,508 | 0,035 | 0,325 | 0,086 | 0,132 | 0,047 | 15 | 6.0324 | 0.139 | 1.3008 | 0.3456 | 0.5272 | 0.1898 |
| 30 | 0,837 | 0,024 | 0,044 | 0,284 | 0,058 | 0,039 | 30 | 1.673 | 0.048 | 0.089 | 0.567 | 0.117 | 0.077 |
| 31 | 1,167 | 0,007 | 0,038 | 0,212 | 0,028 | 0,010 | 15 | 4.666 | 0.027 | 0.152 | 0.848 | 0.111 | 0.038 |
| 32 | 1,023 | 0,015 | 0,023 | 0,029 | 0,050 | 0,018 | 15 | 4.094 | 0.059 | 0.090 | 0.117 | 0.200 | 0.073 |
| 33 | 0,843 | 0,014 | 0,094 | 0,028 | 0,051 | 0,007 | 25 | 2.023 | 0.035 | 0.225 | 0.067 | 0.122 | 0.018 |
| 34 | 0,723 | 0,127 | 0,349 | 0,037 | 0,034 | 0,204 | 45 | 0.965 | 0.169 | 0.465 | 0.049 | 0.045 | 0.272 |
| 35 | 1,035 | 0,008 | 0,119 | 0,025 | 0,023 | 0,007 | 20 | 3.105 | 0.025 | 0.357 | 0.074 | 0.070 | 0.022 |
| 36 | 0,844 | 0,002 | 0,000 | 0,134 | 0,173 | 0,002 | 40 | 1.266 | 0.003 | 0.000 | 0.202 | 0.260 | 0.003 |
| 37 | 0,937 | 0,014 | 0,146 | 0,087 | 0,155 | 0,133 | 30 | 1.875 | 0.029 | 0.291 | 0.175 | 0.310 | 0.265 |
| 38 | 1,031 | 0,020 | 0,073 | 0,200 | 0,171 | 0,119 | 10 | 6.189 | 0.119 | 0.436 | 1.198 | 1.026 | 0.715 |
| 39 | 0,825 | -0,002 | 0,060 | 0,207 | 0,296 | 0,032 | 25 | 1.979 | -0.004 | 0.145 | 0.496 | 0.710 | 0.076 |
| 40 | 0,805 | 0,002 | 0,094 | 0,147 | 0,166 | 0,050 | 20 | 2.416 | 0.007 | 0.283 | 0.441 | 0.498 | 0.151 |
| 41 | 0,985 | 0,034 | 0,212 | 0,095 | 0,054 | 0,258 | 15 | 3.940 | 0.136 | 0.848 | 0.380 | 0.217 | 1.031 |
| 42 | 0,949 | 0,167 | 0,428 | 0,154 | 0,295 | 0,548 | 25 | 2.277 | 0.400 | 1.028 | 0.370 | 0.709 | 1.315 |
| 43 | 0,857 | 0,014 | 0,009 | 0,027 | 0,016 | 0,026 | 15 | 3.429 | 0.057 | 0.038 | 0.108 | 0.064 | 0.105 |
| 44 | 0,681 | -0,009 | 0,088 | -0,003 | -0,004 | -0,001 | 50 | 0.818 | -0.011 | 0.106 | -0.004 | -0.005 | -0.001 |
| 45 | 0,976 | 0,023 | 0,270 | 0,026 | 0,062 | 0,185 | 25 | 2.343 | 0.056 | 0.649 | 0.063 | 0.148 | 0.443 |
| 46 | 0,872 | 0,023 | 0,045 | 0,079 | 0,126 | 0,061 | 10 | 5.231 | 0.137 | 0.269 | 0.475 | 0.754 | 0.363 |
| 47 | 0,981 | -0,008 | 0,151 | 0,103 | 0,238 | 0,002 | 35 | 1.681 | -0.013 | 0.258 | 0.176 | 0.408 | 0.003 |
| 48 | 0,787 | 0,024 | 0,122 | 0,064 | 0,091 | 0,094 | 75 | 0.630 | 0.019 | 0.097 | 0.051 | 0.073 | 0.075 |
| 49 | 0,850 | -0,008 | 0,017 | 0,029 | 0,040 | 0,025 | 45 | 1.133 | -0.011 | 0.022 | 0.039 | 0.054 | 0.033 |
| 50 | 0,835 | 0,009 | 0,146 | 0,066 | 0,017 | 0,004 | 45 | 1.113 | 0.012 | 0.195 | 0.087 | 0.022 | 0.005 |
| 51 | 0,909 | 0,028 | 0,138 | 0,076 | 0,195 | 0,200 | 40 | 1.364 | 0.043 | 0.207 | 0.114 | 0.292 | 0.300 |
| 52 | 0,786 | -0,016 | 0,025 | 0,052 | 0,062 | -0,008 | 50 | 0.943 | -0.020 | 0.030 | 0.062 | 0.075 | -0.009 |
| 53 | 1,073 | 0,009 | 0,050 | 0,024 | 0,043 | 0,026 | 25 | 2.576 | 0.022 | 0.120 | 0.057 | 0.104 | 0.061 |
| 54 | 0,985 | -0,003 | 0,012 | 0,064 | 0,135 | 0,048 | 40 | 1.478 | -0.005 | 0.019 | 0.096 | 0.202 | 0.072 |
| 55 | 0,331 | -0,012 | -0,010 | 0,001 | 0,001 | -0,011 | 75 | 0.265 | -0.010 | -0.008 | 0.001 | 0.001 | -0.009 |
| 56 | 0,437 | -0,010 | 0,076 | 0,001 | 0,049 | -0,009 | 75 | 0.349 | -0.008 | 0.061 | 0.001 | 0.040 | -0.007 |
| 57 | 0,393 | 0,023 | 0,078 | 0,041 | 0,091 | 0,096 | 75 | 0.314 | 0.018 | 0.062 | 0.033 | 0.073 | 0.077 |
| 58 | 0,861 | -0,013 | -0,008 | 0,081 | 0,116 | 0,016 | 45 | 1.148 | -0.017 | -0.011 | 0.108 | 0.155 | 0.021 |
| 59 | 1,060 | -0,006 | 0,055 | 0,397 | 0,463 | 0,248 | 20 | 3.179 | -0.018 | 0.166 | 1.191 | 1.388 | 0.743 |
| 60 | 0,874 | -0,001 | 0,010 | 0,088 | 0,099 | 0,033 | 40 | 1.311 | -0.001 | 0.015 | 0.131 | 0.148 | 0.049 |
| 61 | 0,586 | -0,011 | -0,013 | 0,017 | 0,010 | -0,004 | 75 | 0.468 | -0.009 | -0.011 | 0.013 | 0.008 | -0.003 |
| 62 | 0,383 | -0,012 | -0,019 | 0,010 | -0,004 | -0,009 | 75 | 0.307 | -0.010 | -0.015 | 0.008 | -0.003 | -0.007 |
| 63 | 0,952 | 0,005 | 0,047 | 0,207 | 0,144 | 0,011 | 40 | 1.428 | 0.008 | 0.070 | 0.310 | 0.216 | 0.017 |
| 64 | 0,889 | 0,011 | 0,025 | 0,036 | 0,060 | 0,035 | 40 | 1.333 | 0.016 | 0.038 | 0.054 | 0.091 | 0.053 |
| NHS 1 | -0.001 | 0.008 | 0.000 | -0.011 | 0.011 | 0.012 | 60 | -0.001 | 0.008 | 0.000 | -0.011 | 0.011 | 0.012 |
| NHS 2 | -0.005 | 0.007 | -0.002 | -0.011 | 0.016 | 0.000 | 60 | -0.005 | 0.007 | -0.002 | -0.011 | 0.016 | 0.000 |
| NHS 3 | -0.006 | 0.008 | -0.003 | -0.016 | 0.006 | 0.007 | 60 | -0.006 | 0.008 | -0.003 | -0.016 | 0.006 | 0.007 |
| NHS 4 | 0.017 | 0.008 | 0.003 | 0.001 | 0.022 | 0.030 | 60 | 0.017 | 0.008 | 0.003 | 0.001 | 0.022 | 0.030 |
| NHS 5 | -0.010 | 0.003 | -0.004 | -0.015 | 0.005 | -0.003 | 60 | -0.010 | 0.003 | -0.004 | -0.015 | 0.005 | -0.003 |
| NHS 6 | -0.010 | 0.014 | -0.002 | -0.017 | 0.001 | 0.004 | 60 | -0.010 | 0.014 | -0.002 | -0.017 | 0.001 | 0.004 |
| NHS 7 | -0.008 | 0.002 | -0.002 | -0.020 | -0.005 | -0.001 | 60 | -0.008 | 0.002 | -0.002 | -0.020 | -0.005 | -0.001 |

**TABLE E3:** Amounts of allergen-specific IgE from all allergic patients’ (1-64) and non-allergic individuals’ (NHS1-NHS7) sera included in this study. OD values of the IgE ELISAs specific for rBet v 1.0101, rApi g 1.0101 and the four chimeras were normalized to a substrate incubation period of 1 hour after subtraction of the buffer control values.

|  | **Normalized ELISA OD/h** | | | | | | **OD/h chimeras – OD/h Api g 1** | | | |
| --- | --- | --- | --- | --- | --- | --- | --- | --- | --- | --- |
| **Serum** | **rBet v 1** | **rApi g 1** | **Api-Bet-1** | **Api-Bet-2** | **Api-Bet-3** | **Api-Bet-4** | **Api-Bet-1** | **Api-Bet-2** | **Api-Bet-3** | **Api-Bet-4** |
| 1 | **5.134** | **0.237** | **1.222** | **0.362** | **1.097** | **0.677** | **0.985** | **0.125** | **0.860** | **0.441** |
| 2 | **5.001** | **0.334** | **1.231** | **0.840** | **1.693** | **0.793** | **0.897** | **0.506** | **1.359** | **0.459** |
| 3 | **1.872** | -0.001 | **0.028** | **0.237** | **0.616** | 0.036 | **0.029** | **0.238** | **0.618** | 0.037 |
| 4 | **6.469** | 0.024 | **1.211** | **1.110** | **1.288** | **0.198** | **1.187** | **1.086** | **1.264** | **0.174** |
| 5 | **7.690** | 0.072 | **0.409** | **2.165** | **4.123** | **0.432** | **0.337** | **2.093** | **4.050** | **0.360** |
| 6 | **1.641** | **0.172** | **0.560** | **0.153** | **0.226** | **0.330** | **0.388** | -0.019 | 0.054 | **0.158** |
| 7 | **3.651** | 0.029 | **0.757** | **0.451** | **0.429** | 0.055 | **0.728** | **0.422** | **0.400** | 0.026 |
| 8 | **0.675** | **0.024** | **0.038** | **0.044** | **0.056** | **0.076** | **0.014** | 0.020 | **0.031** | **0.052** |
| 9 | **2.106** | **0.283** | **0.787** | **0.354** | **0.703** | **0.767** | **0.504** | **0.071** | **0.420** | **0.484** |
| 10 | **0.893** | 0.003 | **0.337** | **0.073** | **0.141** | 0.013 | **0.334** | **0.071** | **0.139** | 0.010 |
| 11 | **2.032** | 0.028 | **0.187** | **0.195** | 0.041 | 0.051 | **0.159** | **0.167** | 0.013 | 0.023 |
| 12 | **15.532** | **0.461** | **2.844** | **0.316** | **0.970** | **0.964** | **2.383** | *-0.146* | **0.509** | **0.502** |
| 13 | **1.284** | **0.028** | **0.023** | **0.135** | 0.043 | 0.049 | -0.005 | **0.108** | 0.016 | 0.021 |
| 14 | **1.857** | **0.134** | **0.137** | **0.152** | **0.322** | **0.400** | 0.002 | 0.018 | **0.187** | **0.266** |
| 15 | **5.489** | 0.037 | 0.060 | **0.434** | **0.752** | 0.069 | 0.023 | **0.398** | **0.715** | 0.032 |
| 16 | **6.868** | **0.105** | **2.334** | **0.403** | **0.714** | **0.792** | **2.230** | **0.298** | **0.610** | **0.688** |
| 17 | **8.953** | **0.330** | **1.918** | **0.409** | **1.039** | **0.718** | **1.588** | 0.080 | **0.709** | **0.388** |
| 18 | **1.090** | **0.052** | **0.169** | **0.170** | **0.249** | **0.134** | **0.117** | **0.118** | **0.197** | **0.082** |
| 19 | **2.922** | 0.009 | **0.519** | **0.138** | 0.046 | 0.010 | **0.509** | **0.129** | 0.037 | 0.001 |
| 20 | **4.270** | **0.070** | **1.309** | **0.451** | **0.495** | **0.423** | **1.239** | **0.381** | **0.424** | **0.353** |
| 21 | **2.195** | **0.083** | **0.380** | **0.328** | **0.596** | **0.260** | **0.297** | **0.245** | **0.513** | **0.177** |
| 22 | **2.702** | -0.009 | -0.013 | **0.381** | **0.255** | -0.002 | -0.003 | **0.391** | **0.265** | 0.008 |
| 23 | **2.254** | 0.005 | **0.133** | **0.142** | 0.026 | -0.005 | **0.128** | **0.137** | 0.021 | -0.010 |
| 24 | **2.263** | **0.083** | **0.376** | **0.308** | **0.731** | **0.401** | **0.293** | **0.225** | **0.648** | **0.317** |
| 25 | **1.027** | **0.028** | **0.024** | 0.006 | 0.046 | 0.008 | -0.004 | -0.022 | 0.018 | -0.020 |
| 26 | **1.538** | **0.024** | **0.041** | **0.158** | **0.308** | 0.052 | 0.017 | **0.133** | **0.284** | 0.028 |
| 27 | **2.355** | 0.022 | **0.388** | **0.275** | **0.498** | **0.162** | **0.366** | **0.253** | **0.475** | **0.140** |
| 28 | **3.135** | **0.057** | **0.339** | **0.498** | **0.828** | **0.136** | **0.282** | **0.441** | **0.771** | 0.079 |
| 29 | **6.0324** | **0.139** | **1.3008** | **0.3456** | **0.5272** | **0.1898** | **1.162** | **0.207** | **0.388** | 0.051 |
| 30 | **1.673** | **0.048** | **0.089** | **0.567** | **0.117** | 0.077 | **0.041** | **0.520** | **0.069** | 0.030 |
| 31 | **4.666** | 0.027 | **0.152** | **0.848** | 0.111 | 0.038 | **0.124** | **0.820** | 0.084 | 0.011 |
| 32 | **4.094** | 0.059 | **0.090** | 0.117 | **0.200** | 0.073 | **0.031** | 0.058 | **0.141** | 0.014 |
| 33 | **2.023** | 0.035 | **0.225** | 0.067 | **0.122** | 0.018 | **0.190** | 0.033 | **0.088** | -0.017 |
| 34 | **0.965** | **0.169** | **0.465** | 0.049 | 0.045 | **0.272** | **0.296** | *-0.120* | *-0.124* | **0.103** |
| 35 | **3.105** | 0.025 | **0.357** | 0.074 | 0.070 | 0.022 | **0.332** | 0.050 | 0.045 | -0.003 |
| 36 | **1.266** | 0.003 | 0.000 | **0.202** | **0.260** | 0.003 | -0.004 | **0.198** | **0.257** | 0.000 |
| 37 | **1.875** | 0.029 | **0.291** | **0.175** | **0.310** | **0.265** | **0.263** | **0.146** | **0.281** | **0.236** |
| 38 | **6.189** | **0.119** | **0.436** | **1.198** | **1.026** | **0.715** | **0.317** | **1.079** | **0.907** | **0.596** |
| 39 | **1.979** | -0.004 | **0.145** | **0.496** | **0.710** | 0.076 | **0.148** | **0.500** | **0.713** | 0.080 |
| 40 | **2.416** | 0.007 | **0.283** | **0.441** | **0.498** | **0.151** | **0.276** | **0.434** | **0.491** | **0.144** |
| 41 | **3.940** | **0.136** | **0.848** | **0.380** | **0.217** | **1.031** | **0.712** | **0.244** | 0.081 | **0.895** |
| 42 | **2.277** | **0.400** | **1.028** | **0.370** | **0.709** | **1.315** | **0.628** | -0.030 | **0.309** | **0.915** |
| 43 | **3.429** | 0.057 | **0.038** | 0.108 | 0.064 | 0.105 | -0.019 | 0.051 | 0.007 | 0.048 |
| 44 | **0.818** | -0.011 | **0.106** | -0.004 | -0.005 | -0.001 | **0.117** | 0.008 | 0.006 | 0.010 |
| 45 | **2.343** | **0.056** | **0.649** | 0.063 | **0.148** | **0.443** | **0.593** | 0.008 | **0.092** | **0.387** |
| 46 | **5.231** | **0.137** | **0.269** | **0.475** | **0.754** | **0.363** | **0.132** | **0.338** | **0.617** | **0.226** |
| 47 | **1.681** | -0.013 | **0.258** | **0.176** | **0.408** | 0.003 | **0.272** | **0.190** | **0.422** | 0.016 |
| 48 | **0.630** | **0.019** | **0.097** | **0.051** | **0.073** | **0.075** | **0.078** | **0.032** | **0.054** | **0.056** |
| 49 | **1.133** | -0.011 | **0.022** | 0.039 | **0.054** | 0.033 | **0.033** | 0.050 | **0.065** | 0.044 |
| 50 | **1.113** | 0.012 | **0.195** | **0.087** | 0.022 | 0.005 | **0.184** | **0.076** | 0.011 | -0.006 |
| 51 | **1.364** | **0.043** | **0.207** | **0.114** | **0.292** | **0.300** | **0.164** | **0.071** | **0.250** | **0.258** |
| 52 | **0.943** | -0.020 | **0.030** | **0.062** | **0.075** | -0.009 | **0.049** | **0.081** | **0.094** | 0.010 |
| 53 | **2.576** | 0.022 | **0.120** | 0.057 | **0.104** | 0.061 | **0.098** | 0.035 | **0.082** | 0.040 |
| 54 | **1.478** | -0.005 | **0.019** | **0.096** | **0.202** | **0.072** | **0.023** | **0.101** | **0.207** | **0.076** |
| 55 | **0.265** | -0.010 | -0.008 | 0.001 | 0.001 | -0.009 | 0.002 | 0.011 | 0.011 | 0.001 |
| 56 | **0.349** | -0.008 | **0.061** | 0.001 | **0.040** | -0.007 | **0.069** | 0.009 | **0.048** | 0.001 |
| 57 | **0.314** | **0.018** | **0.062** | **0.033** | **0.073** | **0.077** | **0.044** | **0.015** | **0.055** | **0.059** |
| 58 | **1.148** | -0.017 | -0.011 | **0.108** | **0.155** | 0.021 | 0.006 | **0.125** | **0.172** | 0.039 |
| 59 | **3.179** | -0.018 | **0.166** | **1.191** | **1.388** | **0.743** | **0.184** | **1.209** | **1.407** | **0.762** |
| 60 | **1.311** | -0.001 | **0.015** | **0.131** | **0.148** | 0.049 | **0.016** | **0.132** | **0.149** | 0.050 |
| 61 | **0.468** | -0.009 | -0.011 | **0.013** | 0.008 | -0.003 | -0.002 | **0.022** | 0.017 | 0.006 |
| 62 | **0.307** | -0.010 | -0.015 | **0.008** | -0.003 | -0.007 | -0.006 | **0.018** | 0.007 | 0.003 |
| 63 | **1.428** | 0.008 | **0.070** | **0.310** | **0.216** | 0.017 | **0.062** | **0.303** | **0.208** | 0.009 |
| 64 | **1.333** | 0.016 | **0.038** | 0.054 | **0.091** | 0.053 | **0.022** | 0.038 | **0.075** | 0.037 |
| NHS 1 | -0.001 | 0.008 | 0.000 | -0.011 | 0.011 | 0.012 |  |  |  |  |
| NHS 2 | -0.005 | 0.007 | -0.002 | -0.011 | 0.016 | 0.000 |  |  |  |  |
| NHS 3 | -0.006 | 0.008 | -0.003 | -0.016 | 0.006 | 0.007 |  |  |  |  |
| NHS 4 | 0.017 | 0.008 | 0.003 | 0.001 | 0.022 | 0.030 |  |  |  |  |
| NHS 5 | -0.010 | 0.003 | -0.004 | -0.015 | 0.005 | -0.003 |  |  |  |  |
| NHS 6 | -0.010 | 0.014 | -0.002 | -0.017 | 0.001 | 0.004 |  |  |  |  |
| NHS 7 | -0.008 | 0.002 | -0.002 | -0.020 | -0.005 | -0.001 |  |  |  |  |

bold: significantly positive values

italics: ODs significantly lower than Api g 1-specific ODs

**FIGURE LEGENDS**

**Figure E1. A.** CD-spectra of rBet v1.0101, rApi g 1.0101 and the four chimeric proteins. Spectra are presented as mean residue molar ellipticity. **B.** Comparison of the CD-spectra of Api-Bet-2 and 3 purified at native and denaturing conditions.

**Figure E2.** Binding of the Bet v 1-specific monoclonal antibodies BIP 1 and BIP 4 to rBet v 1.0101, rApi g 1.0101 and the chimeras. Antibody binding was measured by ELISA and detected by an alkaline phosphatase-labeled anti-mouse antibody.

**Figure E3.** IgE binding of 64 sera to rBet v 1.0101, rApi g 1.0101 and the chimeras obtained by ELISA. Horizontal bars, boxes and whiskers indicate medians, quartiles and total ranges, respectively. The distributions of OD-values were compared using the Friedman test (*** P < 0.001, ** P<0.01).

**Figure E4.** Correlation of the amount of Bet v 1-specific IgE with the number of chimeras recognized. OD values obtained by IgE-ELISA were normalized to serum dilutions of 1:10 and alkaline phosphatase substrate incubation times of 1 hour. Bars and whiskers represent medians and quartiles.

**Figure E5.** IgE cross-inhibition between the chimeric allergens. **A.** Direct ELISA; **B.** Inhibition ELISA. Inhibiting chimeras were coated to the solid phase at 1 µg/ml and incubated with patients’ sera. The supernatants were transferred to a second plate with all four chimeras immobilized, and bound IgE was detected by an alkaline phosphatase-labeled anti-human IgE antibody. n.d.: not done
