# Supplementary figures and images for: Chimeras of Bet v 1 and Api g 1 reveal heterogeneous IgE responses in patients with birch pollen allergy
Source: J Allergy Clin Immunol. 2014 Jul;134(1):188–94. doi: 10.1016/j.jaci.2013.12.1073 (PMC4085476; doi:10.1016/j.jaci.2013.12.1073)

**A**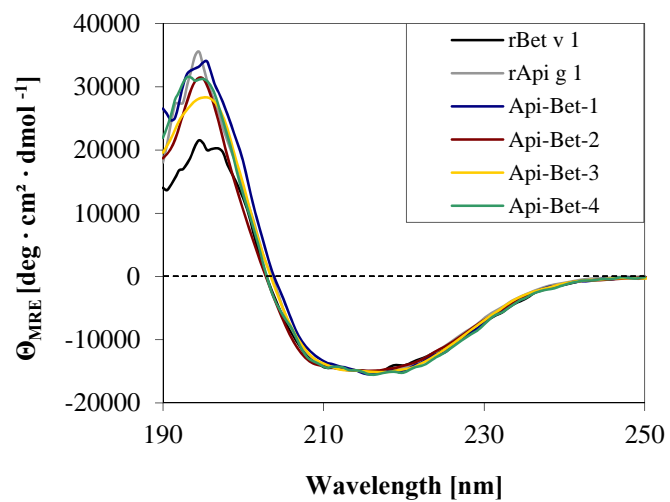**B**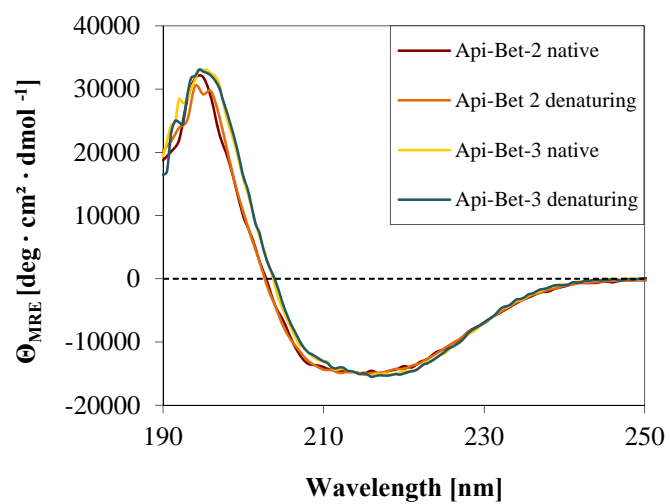

Supplement: Fig E1 [file mmc2.pdf]

**BIP 1**

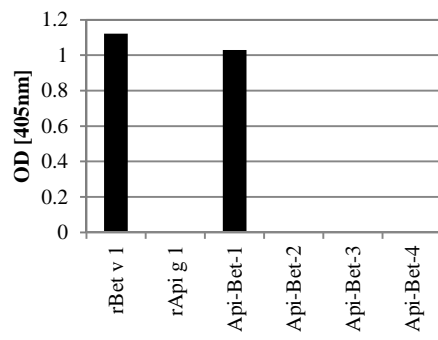

**BIP 4**

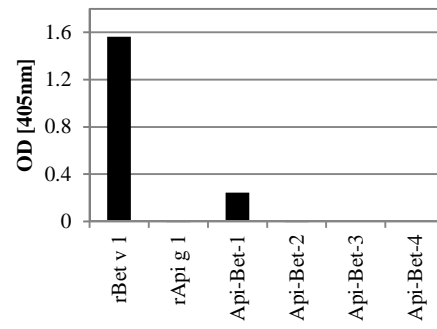

Supplement: Fig E2 [file mmc3.pdf]

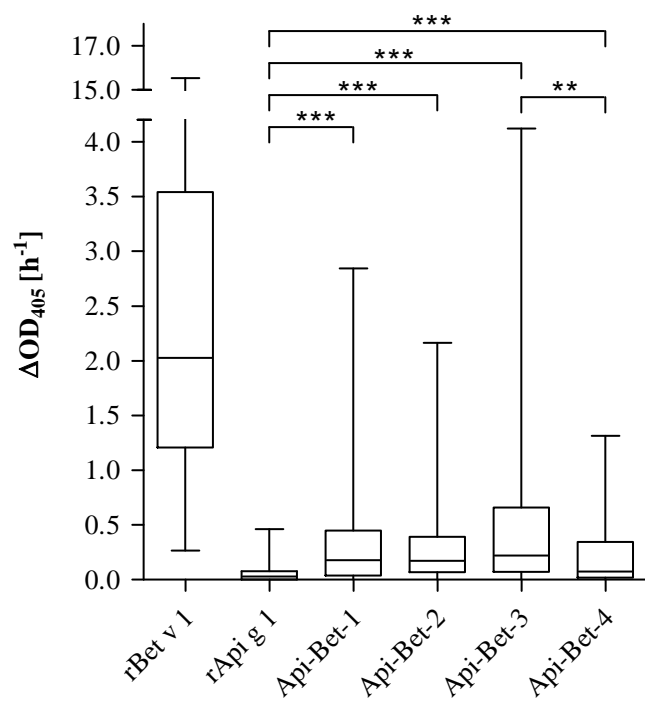

Supplement: Fig E3 [file mmc4.pdf]

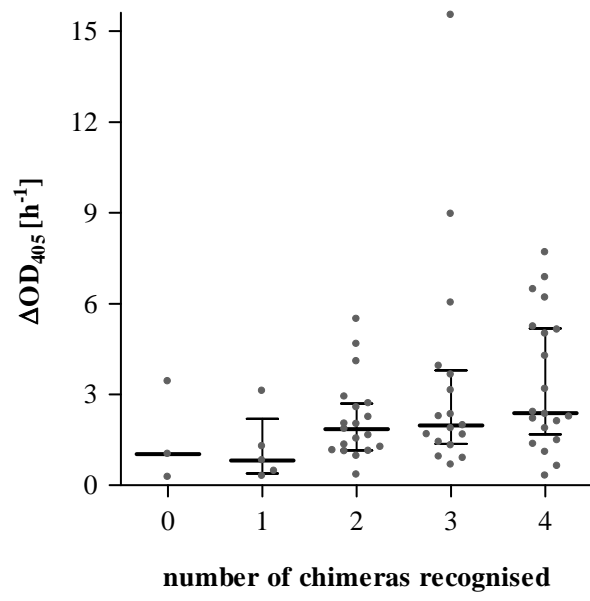

Supplement: Fig E4 [file mmc5.pdf]

**A****Serum 16**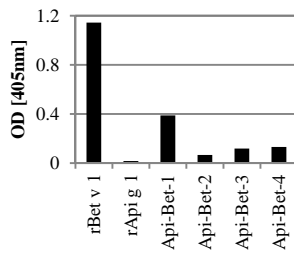**Serum 17**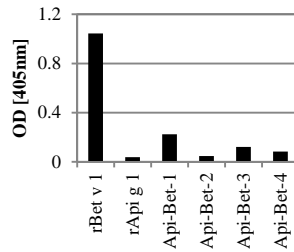**Serum 30**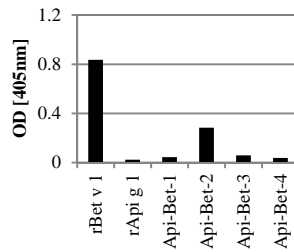**Serum 41**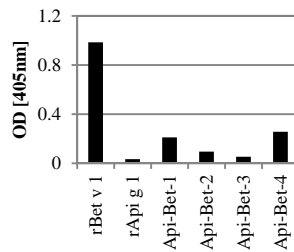**Serum 47**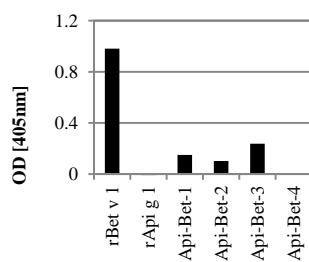**B**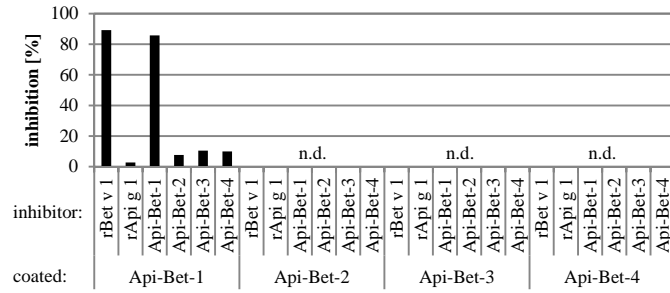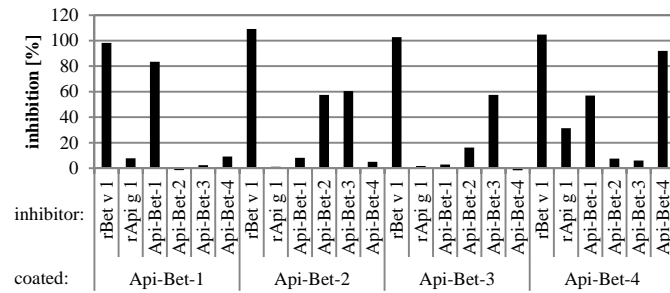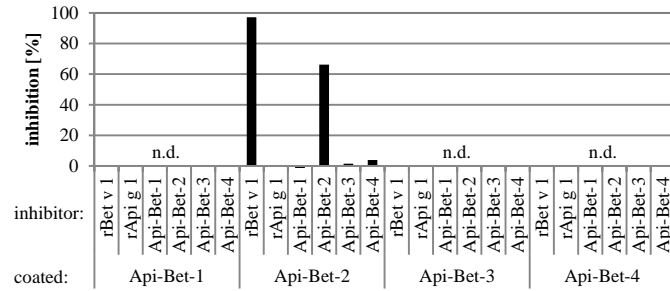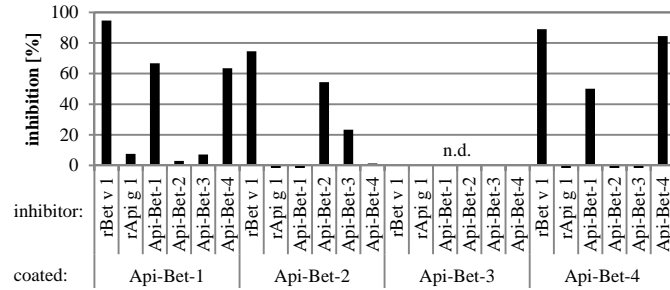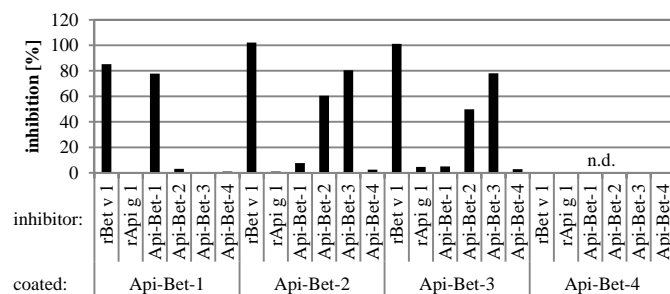

Supplement: Fig E5 [file mmc6.pdf]
